# The Ethics of Teaching Physicians Electronic Fetal Monitoring: And Now for the Rest of the Story *

**DOI:** 10.1055/s-0037-1599229

**Published:** 2017-03-20

**Authors:** Thomas P. Sartwelle, James C. Johnston, Berna Arda

**Affiliations:** 1 Deans and Lyons, LLP, Houston, Texas; 2 Private Practice, San Antonio, Texas; 3 Global Neurology Consultants, Auckland, New Zealand; 4 Department of Medical Ethics, University of Ankara, Ankara, Turkey

**Keywords:** electronic fetal monitoring, cerebral palsy, medical ethics, medical education

## Abstract

Electronic fetal monitoring (EFM) does not predict or prevent cerebral palsy (CP), but this myth remains entrenched in medical training and practice. The continued use of this ineffectual diagnostic modality increases the cesarean section rate with concomitant harms to mothers and babies alike. EFM, as it is used in defensive medical practice, is a violation of patient autonomy and raises serious ethical concerns. This review addresses the need for improved graduate medical education so that physicians and medical residents are taught both sides of the EFM–CP story.

Three decades ago, Dr. John Freeman, editor of the then largest and first ever methodical study on causes of cerebral palsy (CP) and other neurologic birth maladies, 2 warned physicians that the study's research proved that physicians were teaching myth, not science: “If we believe that we should teach only what we know to be true, as opposed to what we know to be myth, then much of what we ‘know’ about pre- and perinatal causes of cerebral palsy (CP), mental retardation (MR) and epilepsy should no longer be taught. . . .[M]any of our assumptions about the factors associated with brain disorders, such as CP, MR and epilepsy, remain rooted in outdated knowledge.” 2

That same warning should be given today about what is being taught to family medicine residents and other physicians-in-training regarding electronic fetal monitoring (EFM). 3 EFM is used in the world's industrialized countries in a great majority of births based on the unproven assumption that EFM can predict and prevent CP by providing caregivers a window of opportunity in which to intervene in labor—mostly by cesarean section (C-section)—and rescue the fetus before irreversible brain damage occurs. 4 5 6 7 8 9 10 11 12 13 14 15 16 This erroneous concept is rooted and grounded in a 19th century birth myth, still prevalent today, that CP and other neurologic birth maladies are caused by oxygen deprivation during labor or delivery. 5 6 7 8 9 10 11 12 13 14 15 16 EFM was conceived in the 1960s based on this birth myth, and that myth is still EFM's foundation today despite decades of research revealing that CP is rarely caused solely by asphyxia, that fetal heart rate is an indirect and poor measure of past and present fetal brain function and damage, that EFM's positive signals are frequently misinterpreted and are wrong 99 times out of 100, and that EFM has caused more harm than good for mothers and babies through unnecessary C-sections. 5 6 7 8 9 10 11 12 13 14 15 16 These facts have been known to medicine for decades. 4 6 7 8 9 10 11 12 13 14 15 16 17 18 Yet the myth is still taught today as gospel in the form of EFM. And EFM continues to be used in 85% of all deliveries in the United States 3 and equally high percentages of births in other countries. 4

Teaching myth and illusion as fact is as prevalent in medicine today as it was when Dr. Freeman penned his warning. As it turns out, medicine in general, not just obstetrics, seems based as much on theories, personal experience, biases, and myth as on empirical evidence. 19 20 21 Studies suggest that almost half the established medical practices constituting core medical care are wrong. 19 20 21 But being wrong is one thing. Causing harm to patients with a scientifically dubious machine like EFM while continuing to teach residents that EFM is efficacious is a failure as breathtaking as medicine's rejection of Semmelweis' simple handwashing cure for childbed fever.

One of the primary reasons EFM has been so widely used is that it is thought to protect doctors and hospitals from CP lawsuits. 4 6 7 9 11 13 15 This is another birth myth, 4 6 7 9 11 13 15 but nevertheless that defensive medicine concept has been passed on from teacher to student since the first CP lawsuits were filed 40 years ago. 6 15 And now most obstetricians in addition to litigation fears have ingrained personal ‘EFM is beneficial’ beliefs or experience that no amount of data will overcome.

EFM defensive medicine causing harm to mothers and babies is an unacceptable ethical mis-step that should have been condemned by the worldwide birth-related professional organizations (BRPOs) and bioethicists long ago, but never has been. 6 15 Their silence, continuing even today, is deafening. So too is the willful blindness of those who continue teaching family and other physicians-in-training to use EFM without mentioning the myriad iniquitous wrongs perpetuated by EFM's continued use under the guise that it is a useful medical modality that can improve fetal outcomes. 3

The rest of the EFM story must be told to physicians-in-training, but especially to mothers in labor, from whom EFM truth has been hidden for almost 50 years. That truth is simple—birth can be a dangerous journey in some cases, and EFM does not help.

## Beginning of Electronic Fetal Monitoring

EFM began as an attempt to mechanize the counting of fetal heartbeats and to coordinate what was thought to be a normal range of heartbeats with contractions. 14 18 22 23 24 25 The idea that fetal heartbeats reflected fetal brain reaction to asphyxia arose from Little's observations published in the 1850s. 14 18 22 Thus, counting fetal heartbeats became the standard of care for birth, and when heartbeats were out of norm, fetal rescue ensued at first with forceps and maneuvers and later with C-sections. 14 18 22

In the 1950s, Yale physician Edward Hon sought to mechanize counting the fetal heartbeats because it was found that a human's ability to count heartbeats by auscultation was not accurate. 6 22 23 24 25 Introduced before the era of evidence-based medicine, EFM never underwent any clinical trials. Hon simply introduced his machine into clinical practice in the late 1960s amid great hope and wonderful paeans about how EFM would defeat CP and other neurologic birth maladies. 6 22 23 24 25 Undisclosed, however, were Hon's and his colleagues' conflict of interest in the ownership of the company manufacturing and selling EFM machines. 6 22 23 24 25 When EFM was finally subjected to randomized controlled trials and scientific scrutiny beginning in 1976, it was found to be no better than intermittent auscultation but also prompted significantly more C-sections, with the increased risks to mothers and babies from that major abdominal operation. 5 6 7 8 9 10 11 12 13 14 15 16 22 23 24 25 26 27 28 29 30 31 32 33 Importantly, in the 50 years since EFM's introduction into clinical practice, the rate of CP has remained the same as when EFM first came on the scene. 4 5 6 7 8 9 10 11 12 13 14 15 16 22 23 24 25 26 27 28 29 30 31 32 33

## A Half Century of Science

When finally subjected to real scientific scrutiny, EFM's defects, faults, and weaknesses resulted in a 50-year-long parade of horribles. EFM clinical trials were first initiated in the 1970s, and from 1976, when the first trial was reported, 22 23 24 25 to 1995, 12 clinical trials found no EFM benefit when compared with intermittent auscultation, but did uniformly find significantly higher EFM C-section rates. 33 34 35 36 37 38 MacDonald, in 1996, analyzed the 12 clinical trials, concluding that abnormal fetal heart rates were not reflective of intrapartum events, but reflected, as Freud observed, neurologic insults early in pregnancy. 34 Although the results of the clinical trials were well publicized, EFM clinical use continued to increase exponentially in labor rooms and courtrooms around the world. 6 11 13 15 27

EFM was proven to have a 99% false-positive rate, 30 and as a test for the absence of injury, it is no better than tossing a coin. 9 EFM does not predict CP, acidemia, neonatal neurologic injury of any kind, stillbirths, or neonatal encephalopathy. 32 More importantly, after 50 years of widespread clinical use, supposed improvements in algorithms, hardware, and assisted technologies 6 12 15 27 39 , exponential numbers of C-sections, and thousands of meetings, task forces, study groups, and statements by BRPOs, there has been no reduction in the incidence of CP or any other neonatal neurologic malady. 4 6 10 27 29 31 32 40 41 42

To a few early observers, it was apparent that EFM was not a monitor, but merely an electronic heartbeat counter. And the recorded information required human interpretation. Interpretation is an art, especially when, as with EFM, there is little scientific data supporting the interpretations. Thus, clinical trials were arranged to test the EFM interpreters. What was found is that the so-called experts frequently disagreed with each other and, more importantly, frequently disagreed with themselves when given the same strip months later. 43 44 45 46 And despite a 50-year effort to improve EFM interpretation, today interpretation remains subjective, impossible to standardize, poorly reproducible, and the inter- and intraobserver contradictions are even more problematic than in the past. 6 12 47 48 49 50 51

EFM has consistently produced significantly more C-sections than any other method of fetal surveillance. In 1970, the C-section rate was 6%. 38 In 2013, 33% in the United States alone and higher in other parts of the world. 4 6 52 53 Contemporary observers ascribe much of the increase to EFM's 99% false-positive rate, 30 defensive obstetrics, and fear of being sued for acting slowly in the face of questionable EFM tracings. 4 6 10 11 13 27 28 29 33 52 54 55 56 57 58 59 60 All involved in obstetrics, except trial lawyers, concede that there is and has always has been a consistent absence of scientific evidence to support the contention that interventions in labor based on any single or combination of EFM patterns prevents CP or any other neurologic impairment. 4 6 10 29 32 52 55 In fact, a prestigious group of maternal fetal medicine scholars publicly acknowledged an evolving maternal fetal medicine consensus that EFM has never had a standard hypothesis related to interpretation and management of supposedly abnormal EFM patterns, and that the time has come to start over and to establish common language, standard interpretation, and reasonable management principles. 29

## Questions

This parade of uncontradicted science obviously raises questions. Why, for almost the last half-century, have physicians continued using a scientifically questionable machine disguised as a safety device that supposedly protects mothers and predicts a baby's future? The partial answer to that question is defensive obstetrical medicine and fear of being sued. 6 8 10 11 13 31 CP myths and the EFM illusions spawned a worldwide litigation crisis centered on the fable that EFM predicts CP and that quick C-section delivery prevents CP and neurologic birth injuries. 6 10 11 12 13 15 27 28 33 This fable is successfully peddled by trial lawyers and their courtroom “experts,” all of whom have a vast profit motive to continue EFM use in every birth. 11 15 27 28 33 This trial lawyer fable has caused physicians to become a CP social welfare scheme, 10 41 which is not only a huge healthcare expense 6 10 11 15 33 41 but also drives caregivers away from obstetrics, 6 10 11 15 33 41 and is the major cause of obstetric defensive medicine—prophylactic, unnecessary medical treatment solely to protect doctors, nurses, and hospitals from lawsuits. 6 10 15 27 33 41

But that question is not especially relevant here. The more important question is why teachers continue to teach residents that EFM use improves fetal outcomes? 3 Even more important than the latter question is why nothing is taught about morbidity and mortality being inflicted on mothers and babies from unnecessary C-sections provoked by EFM's staggering false-positive rate, defensive medicine, and fear of litigation? And, finally, why are residents being taught to use a machine that has no medical benefit to the patient, has the potential to inflict harm, and whose sole function is to protect doctors and hospitals from lawsuits, all without giving mothers informed consent? As it turns out, EFM is a deceitful imposter that desperately needs to be exposed.

## Cesarean Section Deception

Most mothers and the public, and probably many physicians as well, think of C-sections as benign, run-of-the-mill procedures. The worldwide C-section rate 4 6 28 52 56 and the trend toward C-sections on demand 61 illustrate the point. Many C-sections certainly turn out that way. And while C-sections can be life-saving, they are major abdominal surgeries with immediate, significant morbidity and mortality risks—bleeding, infections, embolisms, anesthetic reactions, and surgical injuries to mothers and babies, 15 27 38 52 56 59 as well as significant future risk in subsequent pregnancies—repeat C-sections for life with high rates of operative complications, uterine rupture, and placental abnormalities such as placenta previa and accreta. 52 56 59 62 Recognition of too many EFM-induced and on-demand C-sections has led to articles and workshops in many countries, all with the goal of reducing the number of C-sections. 15 27 33 38 52 56 59 63

But the real risk of too many EFM-induced prophylactic C-sections for protection from lawsuits may not be limited to the known risks. Emerging evidence suggests that C-sections are exposing babies to potential risk of future chronic diseases and neuropsychiatric disorders. 63 64 65 66 67 Despite all these known and emerging negatives, most physicians and hospitals simply required EFM use without giving mothers a choice and without the informed consent required by contemporary bioethics, especially about the dangers of EFM induced C-sections. 15 38 68 69 70 71 72 And residents are given no ethics education related to the daily breach of bedrock ethical principles spawned by EFM use. 3

## Empty Rhetoric

Medical paternalism, the central tenet of the Hippocratic tradition, began to die at almost the exact time EFM came into wide clinical use in 1970. 15 38 73 74 75 Traditional Hippocratic-related ethics were replaced with a radical ethical rethinking called bioethics. 15 38 73 74 75 The central principle of the new deontology was and is patient autonomy—respect for the individual and individual self-determination. 15 38 73 74 75 Critical to self-determination—the individual has the right to choose her medical treatment even if the physician disagrees— 15 38 73 74 75 is the informed consent concept, holding that an individual has the freedom to choose or reject medical treatment based on full, complete information supplied by the physician so that the patient's choice is meaningful. 15 38 73 74 75 Closely associated with autonomy are two other bioethical duties imposed on physicians—beneficence and nonmaleficence. 15 38 73 74 75 Beneficence is the physician's dedication to the patient's welfare, a positive medical goal that differs from nonmaleficence, which is avoiding harm to the patient during medical treatment. 15 38 73 74 75 All three of these foundational ethical principles are violated every day by EFM use and have knowingly been violated for 50 years. 15 38

The right of a patient to be informed about care decisions is a well ingrained fundamental patient right that is expanding under courts' legal enforcement scrutiny in the United States as well as around the world. 76 And while true informed consent is sometimes difficult to apply in practice, 77 78 that is simply not true with EFM. A 9-month relationship would seem more than ample time to allow mothers to discuss and digest EFM's risks and benefits as was pointed out by two consensus EFM task forces more than three decades ago when they recommended mothers be given informed consent before EFM use. 79 80 Obstetricians apparently refused the task forces' advice because nowhere in EFM's almost half-century of existence, among millions of spoken and written words, is there any discussion of giving mothers a choice by telling them the rest of the EFM story, especially the C-section chapter with the discussion of C-section morbidity and mortality risks to mothers and babies. 15 68 69 70 71 72 This same advice has been given by contemporary sources, 31 but so far rejected outright.

Rather than EFM informed consent, physicians choose for the mothers. Physicians choose EFM not because it is a proven medical modality but because they believe it protects them from lawsuits. 6 10 15 27 33 38 Reduced to simplicity, physicians and their BRPOs ignored the published information that EFM was not efficacious, was ineffective, and was in fact harmful; excluded mothers from the EFM risk calculus; and themselves made the decision to use EFM for their self-protection. In modern vernacular, physicians opted for an ethics selfie.

And this ethics selfie comes at a time when BRPOs are beginning to acknowledge EFM's lack of efficacy as well as the fact that 90% or more of CP arises from events other than intrapartum events. 7 31 32 81 Sadly, despite this belated recognition of facts, BRPOs and ethicists alike have failed utterly to recognize the EFM informed consent ethical crisis taking place worldwide every day.

Residency clinical medical ethics education is an important part of any residency program, in particular obstetrics and gynecology, and is part of ACGME (Accreditation Council for Graduate Medical Education) core competencies. 82 EFM would be a textbook ethical teaching tool if only the rest of the story was being told to residents.

## Conclusion

If medicine is concerned that residents learn to use EFM, then medicine must teach the entire story. In fact, teaching all EFM facts would serve a dual purpose. When EFM is exposed in its entirety, bioethics will certainly become a focal point, especially when the physicians-in-training realize that every time a pregnant mother undergoes EFM, she is subjecting herself to the undisclosed risk that false EFM signals will prompt her physician to engage in a self-protection C-section, which, in turn, subjects her and her baby to substantial risks, possibly lifelong risks, associated with that major abdominal surgery. Perhaps then medical paternalism will yield to autonomy, beneficence, and nonmaleficence, and those principles will no longer be mere empty rhetoric.
